# Could Oxidative Stress Regulate the Expression of MicroRNA-146a and MicroRNA-34a in Human Osteoarthritic Chondrocyte Cultures?

**DOI:** 10.3390/ijms18122660

**Published:** 2017-12-08

**Authors:** Sara Cheleschi, Anna De Palma, Nicola Antonio Pascarelli, Nicola Giordano, Mauro Galeazzi, Sara Tenti, Antonella Fioravanti

**Affiliations:** 1 Rheumatology Unit, Azienda Ospedaliera Universitaria Senese, Policlinico Le Scotte, Viale Bracci 1, 53100 Siena, Italy; saracheleschi@hotmail.com (S.C.); annadepalma90@live.it (A.D.P.); pascarelli@unisi.it (N.A.P.); mauro.galeazzi@unisi.it (M.G.); 2 Department of Medical Biotechnologies, University of Siena, Policlinico Le Scotte, Viale Bracci 1, 53100 Siena, Italy; 3 Department of Medicine, Surgery and Neurosciences, Scleroderma Unit, University of Siena, Policlinico Le Scotte, Viale Bracci 1, 53100 Siena, Italy; nicola.giordano@unisi.it; 4 Department of Medicine, Surgery and Neuroscience, Rheumatology Unit, University of Siena, Policlinico Le Scotte, Viale Bracci 1, 53100 Siena, Italy; sara_tenti@hotmail.it

**Keywords:** osteoarthritis, oxidative stress, microRNAs, chondrocytes, taurine

## Abstract

Oxidative stress and the overproduction of reactive oxygen species (ROS) play an important role in the pathogenesis of osteoarthritis (OA). Accumulating evidence has demonstrated the involvement of microRNAs (miRNAs) dysregulation in disease development and progression. In this study, we evaluated the effect of oxidative stress on miR-146a and miR-34a expression levels in human OA chondrocytes cultures stimulated by H_2_O_2_. Mitochondrial ROS production and cell apoptosis were detected by flow cytometry. The antioxidant enzymes SOD-2, CAT, GPx, the transcriptional factor NRF2 and the selected miRNAs were analyzed by qRT-PCR. The H_2_O_2_-induced oxidative stress was confirmed by a significant increase in superoxide anion production and of the apoptotic ratio. Furthermore, H_2_O_2_ significantly up-regulated the expression levels of SOD-2, CAT, GPx and NRF2, and modulated miR-146a and miR-34a gene expression. The same analyses were carried out after pre-treatment with taurine, a known antioxidant substance, which, in our experience, counteracted the H_2_O_2_-induced effect. In conclusion, the induction of oxidative stress affected cell apoptosis and the expression of the enzymes involved in the oxidant/antioxidant balance. Moreover, we demonstrated for the first time the modification of miR-146a and miR-34a in OA chondrocytes subjected to H_2_O_2_ stimulus and we confirmed the antioxidant effect of taurine.

## 1. Introduction

Osteoarthritis (OA) is the most common degenerative joint disorder and it represents the main cause of pain, functional impairment and disability in adult and elderly populations [1]. OA affects the whole joint structure and it is characterized by the progressive degradation of the components of articular cartilage, thickening of subchondral bone and synovial inflammation, inducing the loss of normal joint architecture and function [2].

The release of reactive oxygen species (ROS), such as superoxide anion, hydrogen peroxide (H_2_O_2_) and nitric oxide, contributes to cartilage damage and a concomitant low-grade chronic inflammation [3]. ROS are free radicals containing oxygen molecules derived from cellular oxidative metabolism including enzyme activities and mitochondrial respiration, and play a pivotal role in many cellular functions. Under normal conditions, the production of endogenous ROS is balanced by the antioxidant defence system [4]. ROS have been found to be increased in several pathological conditions, including OA [5]. In OA chondrocytes, ROS overproduction causes DNA damage. Oxidative radical accumulation in the joint contributes to the inhibition of glycosaminoglycan and collagen synthesis and to the activation of metalloproteinases and aggrecanases, promoting cartilage breakdown [5]. It has also been reported that chondrocytes apoptosis related to OA can be induced by oxidative stress [6].

MicroRNAs (miRNAs) are a class of a single-stranded non-coding RNA (20–25 nucleotides in length) involved in post-transcriptional regulation of gene expression by targeting the 3′-untranslated region of the target gene messenger RNA (mRNA) and are implicated in the modulation of several cellular processes and disorders [7]. Accumulating evidence has reported differences in miRNAs expression profiles between normal and OA cartilage samples, demonstrating their role in the development and progression of OA [8,9,10]. Recently, some oxidative stress-responsive miRNAs were identified after the treatment of various cell types with H_2_O_2_ [11]; otherwise, cellular mechanisms regulating oxidative stress were fine-tuned by particular miRNAs [12]. For these reasons, miRNAs seem to be implicated in the articular damage triggered by oxidative stress in OA pathology.

Various agents, such as taurine and ascorbic acid, have been used as potent anti-oxidant substances with highly effective properties attenuating free radical toxicity [13,14]. Data from in vitro studies reported that their antioxidant property could ameliorate ROS-induced cartilage damage [15,16,17]. Taurine (2-aminoethane sulfonic acid) is a necessary amino acid involved in cartilage physiopathology and shows chondroprotective properties principally determining the increase in deposition of extracellular matrix components and in chondrocyte proliferation [18].

In the present study, we investigated the expression levels of miR-34a and miR-146a in human OA chondrocyte cultures stimulated with H_2_O_2_. To evaluate the induced oxidative stress by H_2_O_2_, we performed an analysis of mitochondrial ROS production and cell apoptosis; the gene expression of antioxidant enzymes and of the nuclear factor erythroid 2 like 2 (NFE2L2 or NRF2), the main transcription factor involved in redox homeostasis, which was also detected.

Furthermore, the same analyses were carried out after treatment with taurine, a known antioxidant substance. 

## 2. Results

### 2.1. Mitochondrial •O_2_^−^ Production

**Figure 1:**
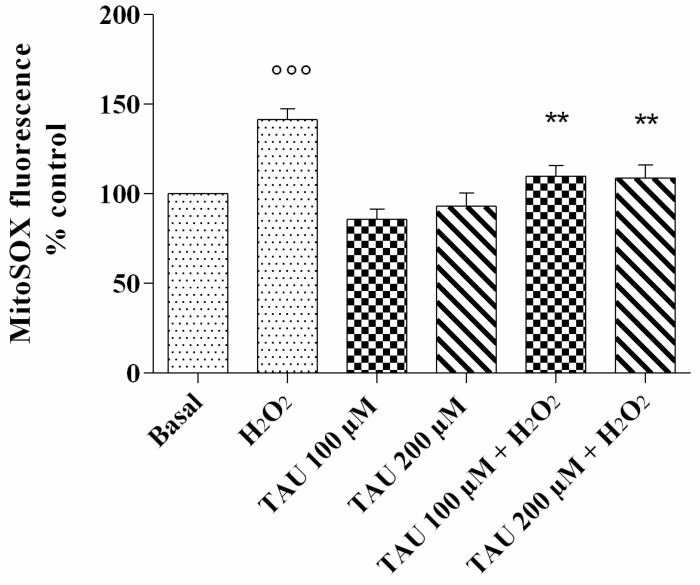
ROS production after 24 h of pre-treatment with taurine (TAU) at the concentrations of 100 μM and 200 μM alone and with the presence of H_2_O_2_ (1 μM) for 30 min. The analysis was performed by using MitoSox Red staining. Data were expressed as a percentage of mitochondrial superoxide anion (•O_2_^−^) production in all the study conditions. The percentage was referenced to the ratio of the value of interest and basal conditions. The value of basal conditions was reported equal to 100. Data were expressed as mean ± SD of triplicate values. °°° *p* < 0.001 versus basal conditions; ** *p* < 0.01 versus H_2_O_2_.

MitoSOX Red staining showed the detection of mitochondrial •O_2_^−^ in chondrocyte cultures after our treatment (Figure 1). As expected, the stimulus of OA chondrocytes with H_2_O_2_ induced a very significant increase in •O_2_^−^ production (*p* < 0.001) in comparison to basal state. No significant modification of •O_2_^−^ release was observed in cells treated with the two concentrations of taurine tested alone compared with basal conditions. The effect of H_2_O_2_ was attenuated and significantly counteracted by the pre-treatment of the cells with taurine 100 μM and 200 μM (*p* < 0.01).

### 2.2. Cell Viability Assay

**Figure 2:**
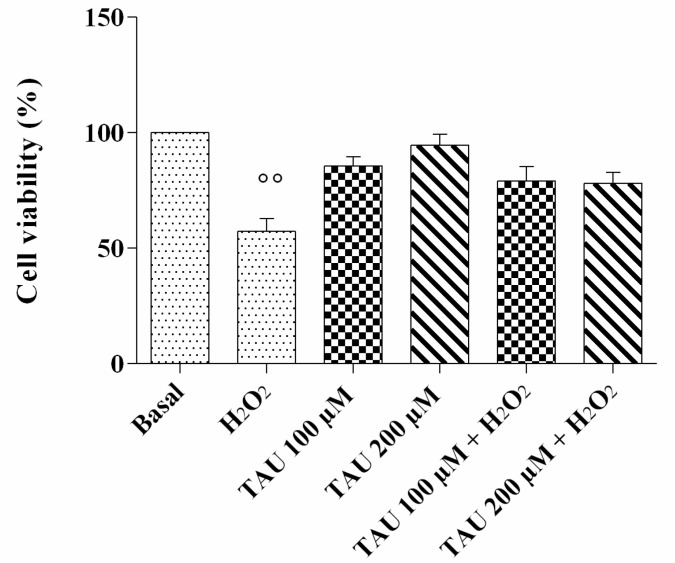
Evaluation of cell viability after 24 h of pre-treatment with taurine (TAU) at the concentrations of 100 μM and 200 μM alone and with the presence of H_2_O_2_ (1 μM) for 30 min. Data were expressed as percentage of cell viability in all the study conditions. The percentage was referenced to the ratio of the value of interest and basal conditions. The value of basal conditions was reported equal to 100. Data were expressed as mean ± SD of triplicate values. °° *p* < 0.01 versus basal conditions.

Cell viability evaluated by (3-[4,4-dimethylthiazol-2-yl]-2,5-diphenyl-tetrazoliumbromide) MTT assay is reported in Figure 2. The chondrocytes exposed to H_2_O_2_ showed a significant decrease in the percentage of survival cells (*p* < 0.01); when cells were treated with taurine tested at concentrations of 100 μM and 200 μM alone or in presence of H_2_O_2_, no significant modifications were observed. The data was confirmed by Trypan Blue test.

### 2.3. Apoptosis Detection

**Figure 3:**
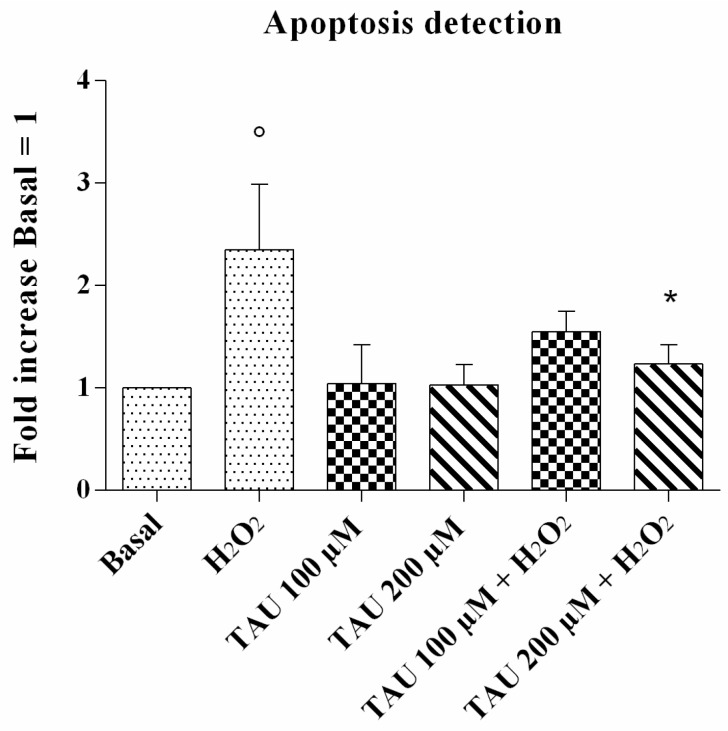
Apoptosis detection after 24 h of pre-treatment with taurine (TAU) at the concentrations of 100 μM and 200 μM alone and with the presence of H_2_O_2_ (1 μM) for 30 min. Apoptosis was measured with Alexa Fluor 488 annexin-V assay. Data were expressed as the percentage of positive cells for Annexin-V and propidium iodide (PI) in all the study conditions. The ratio of apoptosis was referenced to the ratio of the value of interest and basal conditions. The value of basal conditions was reported equal to 1. Data were expressed as mean ± SD of triplicate values. ° *p* < 0.05 versus basal conditions, * *p* < 0.05 versus H_2_O_2_.

The data for chondrocyte apoptosis obtained by flow cytometry assay is reported in Figure 3. The stimulus of chondrocytes with H_2_O_2_ induced a significant increase in this ratio (*p* < 0.05) in comparison to the basal time. The incubation of our cultures with taurine at concentrations of 100 μM and 200 μM did not significantly modify the apoptosis ratio. The increase of apoptosis induced by H_2_O_2_ was significantly reduced by the treatment of the cells with taurine 200 μM (*p* < 0.05).

### 2.4. Modifications of Gene Expression Levels of Factors Involved in Oxidant/Antioxidant Systems

**Figure 4:**
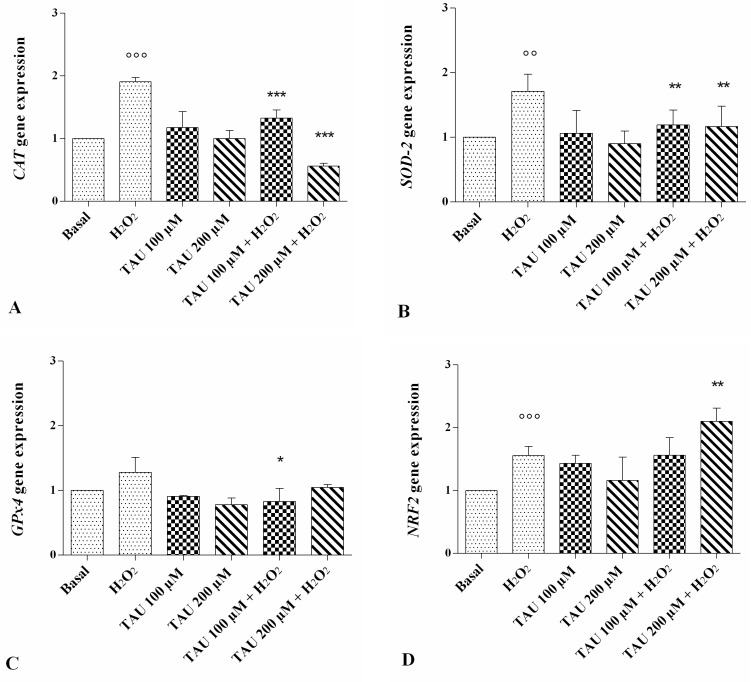
Evaluation of gene expression of antioxidant enzymes catalase (*CAT*) (**A**); superoxide dismutase (*SOD-2*) (**B**); glutathione peroxidase (*GPx*)*-4* (**C**) and of nuclear factor erythroid 2 p45-related factor (*NRF*) *2* (**D**) by real-time PCR. This analysis was performed after 24 h of pre-treatment with taurine (TAU) at the concentrations of 100 μM and 200 μM alone and with the presence of H_2_O_2_ (1 μM) for 30 min. The ratio of gene expression was referenced to the ratio of the value of interest and basal conditions. The value of basal conditions was reported equal to 1. Data were expressed as mean ± SD of triplicate values. °° *p* < 0.01, °°° *p* < 0.001 versus basal conditions, * *p* < 0.05, ** *p* < 0.01, *** *p* < 0.001 versus H_2_O_2_.

The expression levels of catalase (*CAT*), superoxide dismutase (*SOD*)-*2*, glutathione peroxidase (*GPx*)*4* and NRF2 are reported in Figure 4A–D. A statistically significant increase of *CAT* (*p* < 0.001, Figure 4A), *SOD-2* (*p* < 0.01, Figure 4B), and *NRF2* (*p* < 0.01, Figure 4D) expression levels was observed after stimulus with H_2_O_2_. No modification of gene expressions was shown after the incubation of our cultures with taurine 100 μM and 200 μM alone compared with basal conditions. The effect of H_2_O_2_ on *CAT* and *SOD-2* gene expression was significantly counteracted when chondrocytes were treated with taurine 100 μM and 200 μM (*p* < 0.01 for *SOD-2*, *p* < 0.001 for *CAT*). Taurine 200 μM significantly increased the expression levels of *NRF2* (*p* < 0.01) in chondrocytes stimulated with H_2_O_2_. No detectable changes were observed in *GPx4* expression levels after treatment (Figure 4C).

### 2.5. Regulation of miR-146a and miR-34a Gene Expression

**Figure 5:**
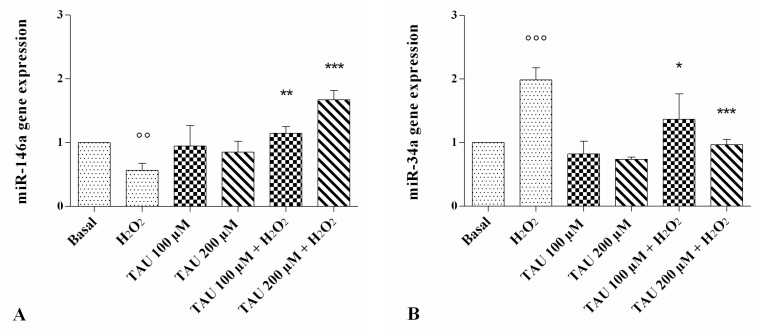
Evaluation of gene expression of miR-146a (**A**) and miR-34a (**B**) by real-time PCR. This analysis was performed after 24 h of pre-treatment with taurine (TAU) at the concentrations of 100 μM and 200 μM alone and with the presence of H_2_O_2_ (1 μM) for 30 min. The ratio of gene expression was referenced to the ratio of the value of interest and basal conditions. The value of basal conditions was reported equal to 1. Data were expressed as mean ± SD of triplicate values. °° *p* < 0.01, °°° *p* < 0.001 versus basal conditions, * *p* < 0.05, ** *p* < 0.01, *** *p* < 0.001 versus H_2_O_2_.

The modification of miR-146a and miR-34a expression levels is represented in Figure 5.

The stimulus of chondrocytes with H_2_O_2_ determined a statistically significant decrease of miR-146a (*p* < 0.01, Figure 5A) and a significant increase of miR-34a (*p* < 0.001, Figure 5B) gene expression. No modification of their expression levels was observed after the incubation of the cells with taurine at both concentrations analyzed alone, compared with the basal time. The stimulus induced by H_2_O_2_ was significantly reversed in chondrocytes pre-treated with taurine at both tested concentrations, inducing an increase in miR-146a and a decrease in miR-34a gene expression.

## 3. Discussion

ROS production is maintained at low levels in healthy articular chondrocytes where they have a role in the activation of intracellular signaling pathways fundamental to the regulation of cartilage homeostasis [4]. Otherwise, the failure in oxidant/antioxidant balance in OA cells determines an altered redox status with an excessive synthesis of ROS, as H_2_O_2_, •O_2_^−^ and hydroxyl free radicals (•OH^−^), contributing to the pathogenesis of OA [5]. 

In the present study, we evaluated the effect of H_2_O_2_-induced oxidative stress on the expressions of some miRNAs responsive to ROS stress. To evaluate the induced stress we performed the analysis of mitochondrial •O_2_^−^ production and of cell apoptosis, as well as the gene expression of antioxidant enzymes and of a transcription factor involved in redox homeostasis.

H_2_O_2_ is often used in in vitro models to mimic the circumstances that drive in vivo oxidative stress cartilage damage [19,20]; in fact, it can diffuse across the outer mitochondrial membrane to the cytosol regulating the activity of ROS sensors and the gene expression of antioxidant enzymes [21,22]. In addition, the exposure of articular chondrocytes to H_2_O_2_ induces the inhibition of proteoglycans synthesis and increases their degradation [23]. 

In our study, the analysis of intracellular ROS content showed upper levels of mitochondrial •O_2_^−^ in chondrocytes stimulated with H_2_O_2_, demonstrating an increase in their production as supported by the data from the literature [24,25]. 

Excessive production of ROS makes chondrocytes more susceptible to oxidant-mediated apoptosis, a major factor in OA pathogenesis [26,27]. H_2_O_2_ influences apoptosis in chondrocytes by changing the intracellular redox state, damaging the structure and function of mitochondria and activating caspase-3 [28]. In accordance with current, state-of-the-art research, our results reported an increase in apoptotic cells after H_2_O_2_ exposure. 

Under normal conditions, the harmful production of endogenous ROS is balanced by the antioxidant defence system including enzymes like SOD-2, CAT and GPx, to decrease oxidative stress. SOD-2 or manganese-dependent (Mn)-SOD represents one of the three SOD family members; it is located in the mitochondrial matrix of the cells and catalyzes the dismutation of superoxide anion in O_2_ and H_2_O_2_, which is then turned in water, to CAT and GPx [29,30]. In vivo and in vitro studies have demonstrated the reduction in expression and activity of these stress-related enzymes in OA chondrocytes, leading to the loss of their ability to scavenge ROS. The resulting oxidation of intracellular and extracellular components contributes to cartilage breakdown in OA. Moreover, the products of cartilage degradation aggravate the inflammation and enhance ROS causing a vicious cycle that contributes to OA progression [31,32]. 

To our knowledge, no studies have been performed to evaluate the response of antioxidant enzymes to H_2_O_2_ in chondrocyte cultures. Conflicting results were reported from studies carried out in different cell types. Dhuna et al. [33] showed the reduced activity of SOD, CAT and GPx enzymes in C6 cells exposed to H_2_O_2_, whereas no modifications in their activities were observed in H_2_O_2_-stimulated Leydig cells in a study by Ding et al. [34]. On the other hand, an increase of SOD-2 expression levels was found in bovine normal chondrocytes cultures after stimulation with IL-1β and IL-6, which are potent proinflammatory cytokines [35].

Our results demonstrated the overexpression of *SOD-2* and *CAT* induced by H_2_O_2_ in OA chondrocyte cultures. The increase of these antioxidant enzymes may be interpreted as a first-line defence against a variety of ROS and proinflammatory stimuli to protect mitochondria against the deleterious effects of free radicals.

The *GPx4* mRNA levels in our cultures appeared unaffected by H_2_O_2_ probably due to the later responsiveness of regulatory elements of *GPx* genes to oxidative stress; analyses carried out at different time points after H_2_O_2_ stimulus could be useful to better investigate this aspect.

The homeostasis of the cellular redox state is also regulated by the transcription factor NRF2. In physiological conditions, NRF2 is maintained at low levels by its ubiquitination and degradation through proteasome machinery. Stress signals lead to its accumulation and its translocation into the nucleus, where it regulates the transcription of antioxidant response element-dependent genes [36]. Its target genes, including *SOD-2*, *CAT* and *GPx*, are involved in the reduction of intracellular levels of ROS, reactive nitrogen species, and electrophiles, protecting cells from oxidative damage [37]. NRF2 may exert a chondroprotective role in OA, suppressing metalloproteinases expression induced by IL-1β [38] and regulating apoptotic cell death [39]. Moreover, results from an in vivo study showed a high susceptibility to OA development in mice with NRF2 deficiency [40]. Our data demonstrated an increase of NRF2 expression levels in chondrocytes stimulated with H_2_O_2_, in agreement with results obtained by Bernard et al. [41] reporting its activation in OA synovial fibroblasts under abnormal ROS signaling. NRF2 response to oxidative stress may contribute to cell survival by enhancing the resistance to further damage stimuli [42]. 

Recently, NRF2 has been found to be regulated by miR-146a, through a miRNA-146a mimic transfection of rat hepatocytes [43]. MiRNA-146a is widely expressed in different species and tissues and plays an important role in immune and inflammatory processes [44]. Its involvement in OA pathogenesis has been investigated recently. Yamasaki et al. [45] demonstrated that miR-146a is upregulated in OA cartilage with a low grade on the Mankin scale compared with normal samples; its expression decreased with the progression of the disease. Furthermore, we reported the reduced expression of this miRNA in OA chondrocytes in comparison to healthy cells in our previous study [46]. Lately, miR-146a has been found to be modulated by H_2_O_2_-induced stress, highlighting its responsiveness to this kind of stimulus [11,12]. The possible modification of miR-146a caused by oxidative stress was investigated by Ji et al. [47], showing a dose-dependent upregulation of the miRNA induced by H_2_O_2_ in PC12 cells. Moreover, MiR-146a overexpression was also observed in HUVEC cells and is associated to the inhibition of proteins responsible for ROS generation, delaying the senescence-like phenotype [48]. On the contrary, we demonstrated the downregulation of miR-146a levels in OA chondrocytes after H_2_O_2_ stimulus. These controversial results could be because of the different responses by various cell types. Indeed, in PC12 cells, the use of antisense-miR-146a, reversed the *SOD-2* expression induced by H_2_O_2_, demonstrating the potential transcriptional regulatory role of the miRNA [47]. Our data, from OA chondrocyte cultures, seems to be in agreement with these results, confirming the interaction between miR-146a and *SOD-2* expression. So, we can speculate about the involvement of oxidative stress in the modulation of miR-146a, which, in turn, regulates the expression of *SOD-2*; these results confirm the role of oxidative stress and miR-146a in the pathogenesis of OA [11,46]. 

Recent evidence shows the contribution of miR-34a to the development and progression of OA; in fact, it has been found to be overexpressed in human OA chondrocytes inducing apoptosis and inhibition of cell proliferation [49]. The use of a miR-34a mimic and anti-miR-34a showed the direct targeting of the miRNA on Sirtuin (SIRT)-1/p53 signaling pathways responsible for the regulation of chondrocyte proliferation and apoptosis during OA damage [49]. Moreover, miRNA profiling analysis identified miR-34a as one of the main regulated miRNAs under oxidative stress [11]. Some studies reported the increase of miR-34a expression levels in human bronchial epithelial and hepatocellular carcinoma cell lines upon treatment with H_2_O_2_ in a concentration-dependent manner [11,50]. To our knowledge, no previous studies have reported the regulation of this miRNA after H_2_O_2_ stimulus in chondrocytes, however, our results appear to be in agreement with the available data showing an increase in miR-34a levels in chondrocyte cultures exposed to H_2_O_2_-induced stress.

Interestingly, Bai et al. [51] found that miR-34a regulated mitochondrial antioxidative enzymes with a concomitant modulation of intracellular ROS levels in primary mesangial cells. Our results showed the regulation of miR-34a expression levels as well as of antioxidative enzymes and intracellular ROS after H_2_O_2_ stimulation in OA chondrocyte cultures. These findings highlight the responsiveness of miR-34a to oxidative stress, and consequently, the possible effect of the miRNA on the oxidant/antioxidant system; on the basis of this evidence, we confirm the involvement of oxidative stress and miR-34a in the pathogenesis of OA. In the current study, we also analysed the role of taurine (100 μM and 200 μM), a well-known antioxidant substance, in counteracting the H_2_O_2_-induced oxidative stress. In our experimental conditions, taurine reduced the mitochondrial •O_2_^−^ production and the ratio of apoptotic chondrocytes in accordance with previous results [52,53,54,55]. Recent evidence demonstrated the increase of antiapoptotic Bcl-2 protein and the reduction of proapoptotic Bax protein expression induced by taurine, confirming our data [56].

Some authors have shown the effect of taurine in preventing oxidant-induced cell damage through the reduction of ROS generation and the neutralization of the pre-existing reactive species [14,56]. Our observation showed the reduction of *SOD-2* and *CAT* expression levels in H_2_O_2_-stimulated chondrocyte cultures pre-treated with taurine, confirming the antioxidant property of this substance.

The chondroprotective effects of some antioxidant substances, such as gingerol and wogonin, have been demonstrated in human OA chondrocytes, through the activation of NRF2 signaling pathway [57,58]. No previous data was available regarding the effect of taurine on this transcription factor, so we showed for the first time a significant increase in *NRF2* mRNA levels in the H_2_O_2_-stimulated chondrocyte cultures after treatment with taurine at a concentration of 200 μM.

Furthermore, we evaluated the role of taurine in regulating the gene expression of miR-146a and miR-34a. To our knowledge, this is the first study reporting the modulation of miR-146a gene expression induced by this antioxidant substance in H_2_O_2_-stimulated chondrocyte cultures, underlining the responsiveness of the miRNA to oxidative stress.

On the other hand, the reduced transcription of the miR-34a by antioxidant agents such as resveratrol, statins and genistein in various cell types is well documented [59,60]. Our data appear to be in agreement with the current literature showing that the increase in miR-34a levels in chondrocytes exposed to H_2_O_2_ was reverted by pre-treatment of the cells with taurine, in particular at a concentration of 200 μM.

## 4. Materials and Methods

### 4.1. Cell Culture

OA human articular cartilage was obtained from the femoral heads of five patients (two men and three women) with hip OA as defined by the clinical and radiological American College of Rheumatology criteria [61], undergoing total hip replacement surgery; OA grade ranged from moderate to severe, and cartilage showed typical osteoarthritic changes, such as the presence of chondrocyte clusters, loss of metachromasia, and fibrillation (Mankin degree 3–7) [62]. OA chondrocytes originated from the area adjacent to the OA lesion. The femoral heads were provided by the Orthopaedics Surgery, University of Siena, Italy. The mean age of the patients was 70 years (range, 64–77 years). The ethics committee of the Azienda Ospedaliera Universitaria Senese/Siena University Hospital approved the use of human articular specimens (decision No. 726 of 2007), and the patients signed an informed consent. 

After surgery, the cartilage was aseptically dissected and minced into small pieces. The fragments were washed in (Dulbecco’s Modified Eagle Medium) DMEM with phenol red, containing 2% penicillin/streptomycin solution and 0.2% amphotericin B. The chondrocytes were isolated from the articular cartilage using sequential enzymatic digestion: 0.1% hyaluronidase for 30 min, 0.5% pronase for 1 h, and 0.2% collagenase for 1 h at 37 °C in the wash solution (DMEM + penicillin/streptomycin solution + amphotericin B). The resulting cell suspension was filtered twice using 70-μm nylon meshes, washed and centrifuged for 5 min at 700 g. The Trypan blue viability test identified a 90% to 95% cell survival. Cells were incubated for 2 weeks at 37 °C and 5% CO_2_ in DMEM culture medium containing 10% fetal calf serum (FCS), 200 U/mL penicillin and 200 µg/mL streptomycin. The medium was changed three times per week. The cell morphology was examined daily with an inverted microscope (Olympus IMT-2, Tokyo, Japan) to avoid the dedifferentiation of expanded chondrocytes and to preserve their phenotypic stability.

For each single experiment, a cell culture from a unique donor was used.

### 4.2. Treatment of Chondrocyte Cultures

In the first passage, OA human chondrocytes were seeded in 6-well dishes at a starting density of 6 × 10^6^ cells/well until they became confluent. Taurine (2-amino ethane sulfonic acid, purity ≥ 98% high-performance liquid chromatography) was purchased from Sigma-Aldrich (Milan, Italy). It was first dissolved in dimethylsulfoxide (DMSO, Rottapharm, Monza, Italy) and then it was further diluted in culture medium immediately before treatment to achieve the final concentration required. The final concentration of DMSO during the treatment did not exceed 0.1%, and it had no effect on the growth of the cells. 

The cells were pre-treated for 24 h with taurine at concentration of 100 μM and 200 μM in the presence or in the absence of H_2_O_2_ 1 μM, added for 30 min after the incubation with the compound. 

After the treatment, the media was removed, cleared through centrifugation, and stored at −80 °C and the chondrocytes were immediately processed to perform cell viability assay, real-time PCR and cytometry analysis.

### 4.3. Mitochondrial Superoxide Anion (•O_2_^−^) Production

OA chondrocyte cultures were plated in a density of 8 × 10^4^ cells per well in 12 multi-plates for 24 h in DMEM with 10% FCS. After that, the medium was discarded and the cells were cultured in DMEM with 2% FBS normally used for the treatment (*n* = 3 different experiments). Then, the cells were incubated in Hanks’ Balanced Salt Solution (HBSS) and MitoSOX Red for 15 min at 37 °C in dark, to evaluate mitochondrial superoxide anion (•O_2_^−^) production. MitoSOX was dissolved in DMSO, at a final concentration of 5 µM. Cells were then harvested by trypsin and collected into cytometry tubes and centrifuged at 1500 rpm for 10 min. Besides, cells were resuspended in saline solution before being analyzed by flow cytometry. A density of 1 × 10^4^ cells per assay (a total of 10,000 events) were measured by flow cytometry and data were analyzed with CellQuest software (Version 4.0, Becton Dickinson, San Jose, CA, USA). Results were collected as median of fluorescence (AU) and represented the mean of three independent experiments (mean ± SD).

### 4.4. MTT Assay

Cell viability was evaluated immediately after the treatment by MTT assay. Chondrocytes were incubated for 3 h at 37 °C in a culture medium containing 10% of 5 mg/mL MTT (Sigma-Aldrich, Milan, Italy). After incubation, the medium was discarded and 0.2 mL of DMSO was added to each well to solubilize the formazan crystals. The absorbance was measured at 570 nm by using a microplate reader (BioTek Instruments, Inc., Winooski, VT, USA). A well without cells was used for blank measurement. 

The percentage of cell survival was calculated as follows:

% Survival = (Absorbance of test)/(Absorbance of control) × 100

The experiments were carried out on pre-confluent cell cultures to prevent contact inhibition influencing the results. Data were expressed as OD units per 10^4^ adherent cells.

### 4.5. Detection of Apoptotic Cells

OA chondrocyte cultures were plated in 12-well plates (8 × 10^4^ cells per well) for 24 h in DMEM with 10% FBS. Then, the medium was discarded, and the cells were cultured in DMEM with FBS 2% normally used for the treatment. The positive control was incubated in the presence of staurosporine, at 0.2 µM (Sigma-Aldrich) for 2 h to induce apoptosis. Then, the cells were washed and harvested with trypsin, collected into cytometry tubes, and centrifuged at 1500 rpm for 10 min. The supernatant was removed, and the pellet was resuspended in 100 μL of 1× Annexin-binding buffer; 5 μL of Alexa Fluor 488 annexin-V conjugated to fluorescein (green fluorescence) and 1 μL of 100 μg/mL propidium iodide (PI) working solution were added to each 100 μL of cell suspension (ThermoFisher Scientific, Rodano, Italy). Cells were incubated at room temperature for 15 min in the dark. After incubation, 600 μL of 1 × Annexin-binding buffer was added before being analyzed by flow cytometry. A total of 1 × 10^4^ cells per assay (10,000 events) [63] were measured on a flow cytometer. The obtained data were analyzed with CellQuest software (Becton Dickinson). The evaluation of apoptosis was performed considering staining cells simultaneously with Alexa Fluor Annexin V and PI, allowing the discrimination of intact cells (Alexa Fluor Annexin-V and PI-negative), early apoptotic state (Alexa Fluor Annexin-V-positive and PI-negative) and late apoptosis state (Alexa Fluor Annexin-V and IP positives). Results were expressed as the percentage of positive cells to each dye (total apoptosis) and represented the mean of three independent experiments (mean ± SD).

### 4.6. RNA Isolation and RT-qPCR

Total RNA, including miRNA, was extracted using TriPure Isolation Reagent according to the manufacturer’s instructions (Roche Diagnostics GmbH, Mannheim, Germany) and was stored at −80 °C. The concentration, purity, and integrity of RNA were evaluated by measuring the OD at 260 nm and the 260/280 and 260/230 ratios by Nanodrop-1000 (Celbio, Milan, Italy). The quality of RNA was also checked through electrophoresis on agarose gel (FlashGel System, Lonza, Rockland, ME, USA). Reverse transcription for target genes was performed by using QuantiTect Reverse Transcription Kit (Qiagen, Hilden, Germany), while for miRNA, by using the cDNA miScript PCR Reverse Transcription (Qiagen, Hilden, Germany) according to the manufacturer’s instructions. 

Target genes and miRNAs were analyzed by real-time PCR using, respectively, QuantiFast SYBR Green PCR (Qiagen, Hilden, Germany) and miScript SYBR Green (Qiagen) kits. All qPCR reactions were performed in glass capillaries using a LightCycler 1.0 (Roche Molecular Biochemicals, Mannheim, Germany) with LightCycler Software Version 3.5. The reaction procedure for target genes amplification consisted 5 s at 95 °C, 40 cycles of 15 s at 95 °C and 30 s at 60 °C. In the last step of both protocols, the temperature was raised from 60 °C to 95 °C at 0.1 °C/step to plot the melting curve. miRNA amplification was performed at 95 °C for 15 s for HotStart polymerase activation, followed by 40 cycles of 15 s at 95 °C for denaturation, 30 s at 55 °C for annealing and 30 s at 70 °C for elongation, according to the protocol.

To further analyze the dissociation curves, we visualized the amplicons lengths in an agarose gel to confirm the correct amplification of the resulting PCR products.

For data analysis, the *C*_t_ values in each sample and the efficiencies of the primer set were calculated using LinReg Software [64] and then converted into relative quantities (RQ) and normalized according to the Pfaffl model [65].

Normalization was carried out considering the housekeeping genes as HPRT-1 for target genes and SNORD-25 for miRNAs. These genes were chosen by software geNorm [66] version 3.5.

### 4.7. Statistical Analysis

Three independent experiments were performed and the results were expressed as the mean ± SD of triplicate values for each experiment. Data normal distribution was confirmed by Shapiro-Wilk, D’Agostino & Pearson and Kolmogorov-Smirnov tests.

Real-time PCR were evaluated by one-way (ANOVA) with a Tukey’s Post Hoc test using 2^−ΔΔ*C*^_T_ values for each sample. 

All analyses were performed using the SAS System (SAS Institute Inc., Cary, NC, USA) and GraphPad Prism 6.1. (GraphPad Software, San Diego, CA, USA). A significant effect was indicated by a *p* value < 0.05.

## 5. Conclusions

In conclusion, in this study we observed the effect of H_2_O_2_-induced oxidative stress on the transcriptional levels of the main factors responsible for the maintenance of homeostatic oxidant/antioxidant equilibrium in human OA chondrocyte cultures. 

Furthermore, we demonstrated for the first time, the regulation of miR-146a and miR-34a gene expression in OA chondrocyte cultures stimulated with H_2_O_2_. On the basis of these results, we highlight the responsiveness of miR-146a and miR-34a to the variation of cellular redox state occurring during OA damage.

Moreover, we confirm the role of taurine as an antioxidant compound, able to partially counteract the oxidative stress damage induced by H_2_O_2_. More importantly, we provide new results about its role in modulating the expression levels of the stress-responsive miR-146a and miR-34a.

Additional studies to confirm the direct relationship between the analyzed miRNAs and oxidative changes occurring in OA chondrocytes are required. The protein levels of the analyzed antioxidant enzymes and of the transcriptional factor NRF2 should be also examined to better understand the metabolic activities induced by H_2_O_2_.

The identification of miR-146a and miR-34a target genes could also be interesting to deeper elucidate their mechanism of action. In fact, *SOD-2* and *NRF2* are candidate target genes of these miRNA and possible effectors of their regulative functions in oxidative stress condition.
